# Prevention of cervical and breast cancer mortality in low- and middle-income countries: a window of opportunity

**DOI:** 10.2147/IJWH.S197115

**Published:** 2019-07-03

**Authors:** Silvia de Sanjose, Vivien D Tsu

**Affiliations:** 1 Sexual and Reproductive Health, PATH, Seattle, WA, USA

**Keywords:** screening, self-exam, prevention, impact, cervical cancer, breast cancer

## Abstract

Breast and cervical cancer are the two most common women’s cancers worldwide. Countries have invested for decades in early detection programs for breast and cervical cancer through screening, community education, and opportunistic case detection by health professionals. However, effectiveness in low- and middle-income countries (LMICs) has been limited due to low coverage, insufficient laboratory capacities for diagnosis, health information systems (HIS) that are not designed to track patients or monitor program performance, barriers that inhibit women’s uptake of services, and inadequate treatment options. Even where some screening activities exist, there has not been sufficient attention to ensuring completion of appropriate diagnosis and treatment after women receive a positive screening test result or report symptoms suggesting cervical or breast cancer. Because of this failure to provide adequate follow-up care, these women miss the potential benefit from early detection and have a higher than average risk to develop cancer or progress to more advanced cancer stages that could have been avoided. There are several critical steps in a woman’s journey from good health to elevated cancer risk, then to cancer prevention or diagnosis, and finally to treatment. There is a window of opportunity that extends from the time a positive finding is identified—by a cervical or breast screening test or recognition of a breast abnormality—to the point when cervical precancer treatment is delivered or a treatment plan for diagnosed breast cancer is initiated. Building on existing health systems and adapting measurable, affordable, and culturally acceptable interventions can achieve a lasting impact. If women can successfully navigate this window of opportunity, they can avoid progression to cervical cancer or greatly reduce the need for invasive treatments for breast cancer and improve their chances for survival and improved quality of life. We propose several actions that can lead us on the path towards reduction of this cancer burden.

## Introduction

### Breast and cervical cancer burden

Breast and cervical cancer are the two most common cancers in low- and middle-income countries (LMICs), both sexes combined. Breast cancer represents 27.3% of all cancers in women in LMICs, with an age-standardized incidence rate of 31.3 per 100,000, representing over half a million new cases every year. Nearly half of affected women will die from breast cancer.^1^ Cervical cancer accounts for 15.9% of the total cancer burden in women living in LMICs with around 300,000 new cases every year. The age-standardized incidence rate is 15.9 per 100,000. Over 87% of these women will likely die from the disease.^2^

Although breast cancer has been regarded as a disease of industrialized geographies and cervical cancer as a cancer of low-resource settings, we are now being confronted with a steady increase in breast cancer incidence in the same areas where cervical cancer used to be the predominant cancer. Breast cancer in LMICs is probably the cancer with the highest increase in incidence in recent years, largely attributable to a transition towards westernized lifestyles that include demographic, reproductive, diet, and physical activity changes.^2^

On the other hand, downward trends in cervical cancer incidence are being observed in selected LMICs, particularly in the Americas,^3^ yet rates remain high in many countries, especially for a cancer that could be largely preventable.^4^ The burden of both cancers is high in LMICs where access to advanced treatment is clearly limited^5^ and will be so for many years to come.

How can countries develop strategies that are feasible and affordable? Analysis of the best-value-for-money strategies indicates that vaccination against human papillomavirus (HPV) and cervical screening at least once in a lifetime together with an opportunistic clinical breast exam (CBE) are strategic actions that could reduce the burden of both diseases, if followed by appropriate management.^6^

### Ongoing progress

LMICs have invested—at modest levels—for decades in early detection activities, particularly for pre-cancerous lesions of the cervix, through screening, and occasionally through opportunistic case detection by health professionals of early breast cancer.^7^ However, effectiveness has been limited due to low coverage, insufficient laboratory capacities for diagnosis, health information systems (HIS) that are not designed to track patients or monitor program performance, barriers that inhibit women’s uptake of services, and inadequate treatment options.^8^ Recently, LMICs have been building resources to monitor non-communicable diseases (NCDs) and to control cervical cancer through vaccination.^9^ Few currently have national programs to support screening, but many are moving towards accelerating strategies that will ultimately reduce the burden of cervical disease among unvaccinated women.^10^,^11^ Secondary prevention of breast cancer in LMICs is clearly in its early stages, as access to the best available tool for tumor detection—mammography—is still limited due to its cost and complexity; more basic options like CBE are the only cost-effective approach in LMICs for the time being.^12^,^13^ Although recommendations have been previously presented that integrate the different actors in cancer prevention, the path is clearly unfinished.^6^,^14^,^15^

### Window of opportunity

Even those countries that do offer basic screening services often fail to follow up a positive finding with adequate treatment. Complex follow-up regimens that require multiple visits can inhibit adequate management, while more streamlined approaches that reduce visits and utilize affordable technologies can enhance it. In Peru, for example, CBE may be the best early detection option in rural environments when access to mammograms is limited. Adding ultrasound to CBE and fine needle aspiration (FNA) biopsy may overcome barriers and improve downstaging by reducing visits and providing care closer to where women live.^16^ In Colombia, women involved in breast cancer early detection underwent up to five visits between the initial screening visit and beginning treatment, if breast cancer were diagnosed.^17^ Breast cancer survival rates at 5 years may go from 70% in Ecuador as compared to 90% in the USA; 5-year survival for cervical cancer can be of 23 points difference in Asian cancer registries (ie, Korea 78%, Thailand 55%) or 35 points difference in the few African cancer registries (72% in Algeria, 38% in South Africa).^2^ Differences may be attributable to late-stage diagnosis and to limited access to treatment.^2^,^18^

Many LMICs worldwide have now a cancer plan that includes cervical and breast cancer strategies for screening and early detection, generally on an opportunistic basis.^19^ However, it is important that any new population intervention promoting screening or early detection provide an adequate system for follow-up. For example, HPV screening tests are slowly being introduced in LMICs as national programs.^20^ Women with a positive test may need to go for a triage test and treatment. Organizing these sequential visits may result in a considerable loss to follow-up. This is why a country may decide on optimal solutions of screen and treat in one single visit. Even women who have received treatment should get a follow-up visit a year later to ensure adequate treatment success and program quality. Thus, registration of screening and treatment interventions is necessary for an adequate continuum of care and is rarely available.^21^–^24^

**Figure 1:**
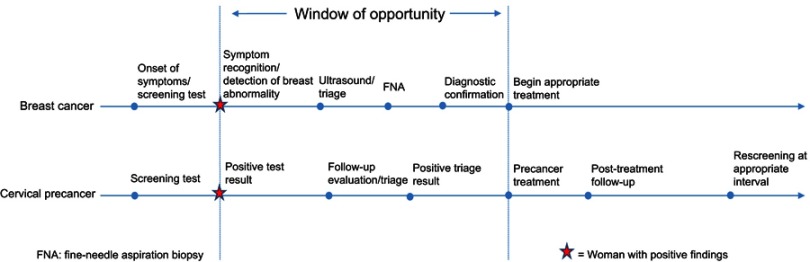
Window of opportunity in continuum of care for breast and cervical cancer prevention.

The point of this paper is to raise awareness of the need to follow those women who, after accessing a secondary prevention exam or reporting potential symptoms, turn out to have a positive test. These women are likely to be at higher risk of cancer than the general population and may require additional tests to confirm diagnosis. At this stage there is a window of opportunity that extends from the time a positive finding is identified—by a cervical or breast screening test or recognition of a breast abnormality—to the point when cervical precancer treatment is delivered or a treatment plan for diagnosed breast cancer is initiated, during which prompt and appropriate intervention can prevent the development of cervical cancer or late stage presentation of breast cancer (Figure 1).^21^

We think that by focusing on four critical areas in a structured manner, countries could gain the necessary momentum to reach vulnerable populations with effective services during this window of opportunity.

1. Identify and track women with a positive screening test or clinical finding of cervical or breast cancer by strengthening the HIS and increase the use of HIS outputs by health workers.

HIS in LMICs are generally designed for program monitoring using aggregated service data—often with different systems for different facility levels and poor or nonexistent linkages between the systems—that makes tracking of individual clients nearly impossible.^25^ The systems often rely on women to manage their own follow-up or depend on individual health care worker initiative to seek out women overdue for recommended care. In a recent WHO review of eHealth challenges in the Americas, PAHO concluded that—despite considerable progress—lack of unique patient identifiers, inadequate mechanisms for exchange of clinical data between systems, and absence of legal frameworks to facilitate data exchange remain as significant problems.^26^ Although several countries, especially in Africa, have adopted electronic platforms like the District Health Information Software 2 (DHIS2), it does not allow longitudinal tracking; in addition, limited human resources for data entry, inadequate training, and equipment limitations can lead to incomplete or incorrect data entry.^25^ Even when using a single-visit approach to cervical cancer screening, traceability is still required for women not eligible for ablative treatment and for confirmation of cure one year afterwards for all treated women.

Assessing the existing data flow contained in each health system and enforcing registration of basic data can facilitate traceability for women needing follow-up. Countries can then determine what changes are needed and whether additional computer-assisted and eHealth solutions are feasible and effective in reaching women needing follow-up.

2. Strengthen the availability and quality of diagnostic and treatment services for breast abnormalities and cervical precancer by evaluating current service availability, increasing capacity where needed, and measuring adherence to quality standards.

Taking the example of Peru, a model program to improve early detection of breast cancer was developed to be a more resource-appropriate alternative to the standard national guidelines and is currently being scaled up.^16^,^27^ In Guatemala and Nicaragua, where HPV testing has been introduced in selected departments, new patient treatment algorithms were implemented to govern triage testing and treatment options after a positive HPV test result.^8^,^28^ However, there is preliminary evidence that different algorithms are being applied, and the quality of screening outcomes is not uniformly good.^29^ There is a need for expert review of these algorithms, their corresponding national guidelines, and how they are being implemented. A careful review can generate the recommendations for adjustments that are needed in practice. Remedial training to address weaknesses can be offered where needed, and innovative quality assurance mechanisms to strengthen capacity and quality can be applied.

3. Identify potential barriers and facilitators affecting traceability, mitigate the barriers, and expand on patient navigation services.

Whenever there is an intervention in asymptomatic populations, it is important to identify barriers that may limit a full uptake of provided services.^30^–^33^ It is important to explore the usefulness, feasibility, and acceptability of interventions and solutions to mitigate barriers, such as use of mobile phones or text messages.^34^–^36^ Patient navigation programs can help to shorten the time from initial presentation to final diagnosis and entry to treatment.^37^ Community health workers are also an important resource that can provide insight into the needs of women and potential misunderstandings of health-related issues.^38^ Workshops including community stakeholders can contribute to tailoring interventions and implementing solutions to reduce the barriers to service uptake.

4. Generate country-specific evidence on the effectiveness of the interventions, costs of inputs, and value for money (cost-effectiveness).

Finally, it is important to evaluate each intervention in terms of value-for-money towards the ultimate goal of reductions in incidence (cervical cancer) and mortality (both cancers). Data on incremental use of follow-up services, health impact, and costs of new interventions can be used to construct disease-outcome models to estimate the value-for-money and the anticipated national budget impact of the different approaches. In-depth evaluation of all process activities (ie, providers person-time at the clinic; resources to introduce data; time and services for follow-up; task shifting) may help the system to refine service flows and eliminate unnecessary costs.^39^

We call for a multidisciplinary and multisectoral approach to this window of opportunity that can ensure that women with breast abnormalities or with a positive cervical screening test receive the full spectrum of care needed to follow-up, clarify their diagnosis, and, if necessary, treat their condition in a timely way.

Interventions to improve traceability, increase access to quality diagnostic and treatment services, and facilitate women’s ability and willingness to complete follow-up care, combined with careful analysis of cost-effectiveness, should be available to all women regardless of residence or socioeconomic status. Building on existing health systems and adapting measurable, affordable, and culturally acceptable modifications can achieve a lasting impact in low-resource settings. If women can successfully navigate this window of opportunity, they can avoid progression to cervical cancer or greatly reduce the need for invasive treatments for breast cancer and improve their chances for survival and improved quality of life.
